# ENGAGING CERTIFIED NURSING ASSISTANTS TO SUPPORT COMMUNICATION FOR PEOPLE LIVING WITH DEMENTIA IN NURSING HOMES

**DOI:** 10.1093/geroni/igad104.1909

**Published:** 2023-12-21

**Authors:** Natalie Douglas

**Affiliations:** Central Michigan University, Mount Pleasant, Michigan, United States

## Abstract

Over 2 million Americans live in nursing homes (NHs), and the vast majority of them live with dementia negatively impacting their ability to communicate. Certified nursing assistants (CNAs) provide the majority of direct care to people living with dementia (PLWD) in NHs, yet they do not have access to person-centered, evidence-based tools to support communication with this population. When CNAs do not communicate with PLWD in effective ways, PLWD may demonstrate negative behavioral symptoms that interfere with care, impacting not only the PLWD, but also the CNA, contributing to CNA turnover and burnout. Most interventions designed to support communication between the PLWD and the CNA do not involve CNA engagement which may contribute to the lack of implementation and access of such interventions. The purpose of this presentation is to outline the process taken to engage CNAs in the implementation of an intervention designed to improve communication during care activities. Information about questions asked to CNAs both in formal (e.g., interviews, focus groups) and informal (e.g., hallway conversations) ways will be provided. Although a sample of CNAs from 6 NHs (n=12) initially rated the intervention as acceptable (M = 4.5, SD = .48), appropriate (M = 4.4, SD = .58), and feasible (M = 4.28, SD = .49) where a rating of ‘5’ equals more acceptable, appropriate, and feasible, and ‘1’ equals less acceptable, appropriate, and feasible, this presentation will highlight how additional levels of engagement prompted modifications to the intervention, supporting implementation.

